# Spin State Switching in Heptauthrene Nanostructure by Electric Field: Computational Study

**DOI:** 10.3390/ijms222413364

**Published:** 2021-12-13

**Authors:** Karol Szałowski

**Affiliations:** Department of Solid State Physics, Faculty of Physics and Applied Informatics, University of Łódź, ulica Pomorska 149/153, 90-236 Łódź, Poland; karol.szalowski@uni.lodz.pl

**Keywords:** heptauthrene, nanographene, Hubbard model, graphene magnetism, magnetic phase diagram, spin switching

## Abstract

Recent experimental studies proved the presence of the triplet spin state in atomically precise heptauthrene nanostructure of nanographene type (composed of two interconnected triangles with zigzag edge). In the paper, we report the computational study predicting the possibility of controlling this spin state with an external in-plane electric field by causing the spin switching. We construct and discuss the ground state magnetic phase diagram involving S=1 (triplet) state, S=0 antiferromagnetic state and non-magnetic state and predict the switching possibility with the critical electric field of the order of 0.1 V/Å. We discuss the spin distribution across the nanostructure, finding its concentration along the longest zigzag edge. To model our system of interest, we use the mean-field Hubbard Hamiltonian, taking into account the in-plane external electric field as well as the in-plane magnetic field (in a form of the exchange field from the substrate). We also assess the effect of uniaxial strain on the magnetic phase diagram.

## 1. Introduction

Electric field control of magnetism lies at heart of the developing spintronics [1]. For this purpose, a variety of materials and a wide range of physical mechanisms have been employed [1,2,3,4], with emphasis put on the nanostructures. A natural route to synthesis of reproducible nanostructures is resorting to molecular systems. As a consequence, an emerging field is molecular spintronics [5,6,7], utilizing molecules for information storage and processing. Another platform attracting significant attention of researchers and boosting hopes for technological progress is graphene, a unique flat material. Development of graphene-based spintronics is a highly promising perspective [8,9,10]. In addition to the unique properties of two-dimensional graphene sheets, various forms of nanographenes (graphene nanoflakes), being actually polyaromatic hydrocarbons [11,12,13,14], constitute potentially promising candidates for the applications in the field of spintronics. Such systems, at the cross-section of physics and chemistry, combine the advantages of molecular systems (such as a chemical route to synthesis of fully reproducible nanostructures) and the unique properties and potential of graphene. In addition, the presence of edge in nanographene offers an additional possibility of modifying a wide range of its properties to reach the desired behaviour.

The constant progress in synthesis of graphene nanostructures with well-defined edges of specific geometry [15,16] invigorates further the interest in studies of the graphene nanostructures. Such progress, resulting in achievement of atomic precision within the bottom-up strategy of synthesis, involves both graphene nanoribbons [17,18] and nanographenes of diverse shape and edge form [19], including triangulene [20,21,22,23], triangulene dimers [24], rhombene [25], hexagons [26], nanostars [27] and triangulene rings [28] or chains [29] as well as doped nanographenes [30]. The atomic precision in shape gives the opportunity to take advantage of geometry-dependent electronic states in the nanostructures. The ability to control the geometry precisely also provides control over the magnetism in graphene nanostructures: the topic which has attracted significant attention since its emergence [31,32,33,34]. One of the most interesting structures are those which exhibit the ground state with nonzero spin [31,34], due to unequal number of carbon atoms belonging to two interpenetrating sublattices (as predicted by Lieb theorem [35] and refined by further works [36]). Gaining control over the geometry of nanographenes paved the way towards effective design and engineering of the magnetic nanographenes with desired properties [26,37] (including the possibility of assembling analogous structures with scanning probe [38]).

In order to gain the control over the nanographene magnetism, the effect of the electric field on the electronic structure of the graphene nanostructures has to be studied. The most desired property is the switching of the total spin of the nanostructure under the influence of the field between non-zero value and zero value. The total spin equal to zero can either correspond to the fully non-magnetic (NM) state or can describe an antiferromagnetic (AFM) alignment of magnetic moments. As a consequence, switching between all the mentioned states can be highly interesting. Numerous computational works aimed at capturing the spin switching effect can be mentioned. First of all, the triangular zigzag-edged graphene nanoflakes attracted significant attention of theorists in various contexts related to their magnetism [32,39,40,41]. This is due to the predicted presence of the edge electronic state exhibiting magnetic polarization (the zigzag edge of graphene [42,43]). As a result, the control over the magnetism in triangular zigzag-edged nanoflakes with electric fields was extensively discussed in Refs. [44,45,46,47,48,49,50]. The phenomenon of magnetic depolarization of a ferromagnetic triangular zigzag-edged graphene nanoflake with a significant electric in-plane field has been investigated in Ref. [51]. The electric field-modified optical properties of triangular zigzag-edged nanostructure were discussed in Ref. [52].

The electric field-controlled magnetic properties of other nanographenes also attracted the attention, including rectangular nanostructures [53,54], armchair-edged systems [55], or bow-tie graphene nanoflake with zigzag edge investigated in Ref. [56] or circular graphene quantum dots in Ref. [57]. Finally, bilayer structures should be mentioned, such as triangular structures [58,59,60], rectangular structures [61,62], or other bilayers [63]. In addition, the electric field-modified optical properties of various graphene nanoflakes were studied in Ref. [64]. Another studied graphene-like systems with electric field-controllable magnetic properties are, for example, stanene nanoribbons [65,66].

However, the fundamental issue from the point of view of nanographene molecular systems application in spintronics is synthesis with atomic precision and undoubtable microscopic confirmation of the presence of magnetically polarized states predicted by the theory. Only such factors permit the progress and motivate further studies of the magnetic phenomena in nanographenes. In the context of spin manipulation in graphene nanostructures, the important experimental results are present in the recent literature. For example, Ref. [67] for nanoribbon-like structures can be mentioned, revealing the Kondo physics emerging due to the existence of localized magnetic moments at the edge of graphene nanostructure. However, even smaller and more regular nanostructures were investigated in this context. The presence of the spin-triplet state, with total spin equal to S=1, has been recently proved and studied experimentally in such molecular nanographenes as triangulene [68] and heptauthrene [69] (the latter one composed of two triangular units).

The results recalled above provide a strong motivation for studying the effect of the electric field on the S=1 state and the possibility of its switching to S=0. In particular, they encourage undertaking the computational predictions of the spin switching possibility in small nanographene molecules synthesized recently. Such studies would guide experimental research of key importance for development of nanographene-based spintronics.

Taking into account the motivation sketched above, in the present paper, we perform computations aimed at prediction of the magnetic phase diagram of heptauthrene nanostructure immersed in in-plane electric field to verify the possibility of spin switching. As a particular result, we find the possibility of switching the spin between S=1 and S=0 value using the electric field of moderate strength and emphasize the importance of the field orientation for this effect. In addition to the S=0 NM state, we predict the presence of AFM state in the nanostructure. The spin density for magnetic states is found to be distributed primarily along the longest zigzag edge of the molecule. In our calculations, we account for the possible presence of magnetic exchange field from the substrate (due to proximity effect), and we characterize its importance for spin switching effect, parallel to the effect of the electric field. We also address the issue of influence of uniaxial strain on the predicted properties.

In the next part of the paper, we describe the theoretical method used for computation of the magnetic properties of heptauthrene. The following section contains the extensive presentation of the numerical results. Finally, we offer discussion and concluding remarks.

## 2. Methods

**Figure 1:**
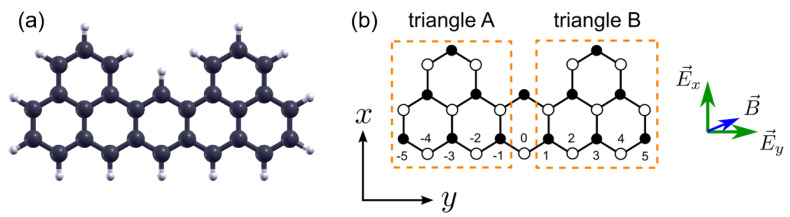
(**a**) View of the full heptauthrene nanostructure, consisting of carbon atoms (larger balls) and hydrogen atoms (smaller balls) [70]; (**b**) schematic view of the heptauthrene nanostructure with carbon atom positions marked with circles. Open and filled circles indicate the majority and minority carbon sublattice, respectively. Two triangles connected with a central dimer of carbon atoms are encircled with dashed lines. The labels of carbon sites at the longest zigzag edge are shown. The directions of in-plane electric fields and in-plane magnetic field are marked.

The schematic view of the heptauthrene nanostructure, being a system of interest in the present work, is shown in Figure 1. The orientation of the external electric field is marked, together with an arbitrary in-plane magnetic field leading to the Zeeman splitting of the energy states (possibly originating from the exchange field due to proximity effect with substrate). Filled and empty circles in Figure 1b denote two graphene sublattices of the nanoflake, denoted by A and B. The structure has unequal number of C atoms in both sublattices, so that $N_{A}=15$ (majority sublattice) and $N_{B}=13$ (minority sublattice). According to Lieb theorem [35], when the electronic spectrum of the structure obeys the half-filling condition, this imbalance gives rise to non-zero spin at the ground state, equal to $S=|N_{A}-N_{B}|/2=1$ (see also Ref. [33]).

The geometry of the heptauthrene nanostructure resembles a pair of interconnected triangular structures. The dashed lines illustrate the division of the nanoflake to such two non-overlapping triangular parts, with the central dimer connecting the triangles left unassigned to any of them (this division is mentioned here just for the purpose of further discussion of magnetizations of both triangles and has no effect on the theoretical modeling of the system, which is treated as a single entity).

In order to describe the magnetism in the presented nanostructure, we employ the formalism based on the Hubbard model incorporating the external electric and magnetic field, and we solve the model within mean-field approximation. Such an approach, built on the grounds of the Hubbard model, was applied in the literature to study the magnetism of geometrically confined graphene [34]. It is remarkable that the good qualitative agreement between the mean-field based model and Quantum Monte Carlo simulations has been found [71]. In addition, the approach was confronted successfully with *ab-initio* calculations [72,73]. The approach has also been confronted with the experimental data regarding the predicted energy gap in nanoribbons [74]. What is even more important is that this model was used to predict the electronic properties of recently synthesized nanographenes, and its outcome was successfully compared to the scanning tunneling microscopy characterization of the nanostructures [22,23,37,68].

The model Hamiltonian takes the following form:

(1) $$\begin{array}{cc} H= & -\sum_{\langle i,j\rangle ,\sigma }^{}t_{i,j}\;\left(c_{i,\sigma }^{†}c_{j,\sigma }+c_{j,\sigma }^{†}c_{i,\sigma }\right)+U\sum_{i}^{}\left(\langle n_{i,↑}\rangle n_{i,↓}+\langle n_{i,↓}\rangle n_{i,↑}\right)-U\sum_{i}^{}\langle n_{i,↑}\rangle \langle n_{i,↓}\rangle \\ & +2\Delta \sum_{i}^{}s_{i}^{z}+eE_{x}\sum_{i,\sigma }^{}x_{i}n_{i,\sigma }+eE_{y}\sum_{i,\sigma }^{}y_{i}n_{i,\sigma }. \end{array}$$

The core of the Hamiltonian is the tight-binding part, which describes electronic hopping between nearest-neighbouring sites denoted by $\langle i,j\rangle$ with hopping energy equal to $t_{i,j}$. For the structure with ideal geometry, all the hopping integrals are taken as equal to t= 2.8 eV. If the presence of the strain is taken into account, the following relation is used [75,76]:

(2) $$t_{i,j}=t\;e^{-\beta \left(\frac{d_{i,j}}{d_{0}}-1\right)},$$

where the exponent β=3 and $d_{i,j}$ is the length of the bond between carbon atoms at nearest-neighbour positions *i* and *j* for given strain (while the value for ideal unstrained geometry is $d_{0}$, assumed to be equal to 1.42 Å). The bond length for bonds extending only along the *x*-direction (armchair direction) is $d_{i,j}=d_{0}\left(1+\varepsilon_{x}\right)$, while, for the remaining bonds (along zig-zag direction), it is $d_{i,j}=\left(d_{0}/2\right)\sqrt{{\left(1+\varepsilon_{x}\right)}^{2}+3{\left(1+\varepsilon_{y}\right)}^{2}}$. In the formulas, $\varepsilon_{x}$ and $\varepsilon_{y}$ stand for the uniaxial strain in the *x*- or *y*-direction, respectively.

The operator $c_{i,\sigma }^{†}\left(c_{i,\sigma }\right)$ creates (annihilates) an electron with spin σ=↑,↓ at site i=1,...,N, where N=28. The further part of the Hamiltonian describes the coulombic interactions between the electrons in the spirit of mean-field Hubbard model, where *U* (taken as U/t= 1.3 after [69]) accounts for the coulombic interaction energy between opposite-spin electrons housed at the same site. The presence of the magnetic field, in a form of the exchange field (originating from the substrate due to the proximity effect [77,78,79,80,81]) acting on *z* components of spins $s^{z}=\left(n_{i,↑}-n_{i,↓}\right)/2$, is introduced by the parameter Δ. The components of the electric field along the *x*- and *y*-directions (see Figure 1) are denoted by $E_{x}$ and $E_{y}$, respectively. The coordinates of the individual sites equal to $x_{i}$ and $y_{i}$ (taking into account the rescaling by the factors $\left(1+\varepsilon_{x}\right)$ and $\left(1+\varepsilon_{y}\right)$ if an uniaxial strain is present), while *e* is the elementary charge. The Hamiltonian resembles the model which we have used in the studies of graphene nanoflakes in the external field [53,61].

The solution of the model is obtained by the self-consistent diagonalization of spin-up and spin-down Hamiltonians (singled out from Equation (1)) and calculation of the average numbers of electrons at all sites, $\langle n_{i,\sigma }\rangle$ (as described in full detail in our Ref. [53]). The half-filling condition for the Hubbard model is accepted, so that the presence of the fixed number of electrons equal to the number of the carbon atoms is assumed. All the spin-up and spin-down electron numbers, $N_{↑}$ and $N_{↓}$, are tried (constrained with the condition $N_{↑}+N_{↓}=N$) to achieve the minimization of the ground-state enthalpy of system $\langle H\rangle$. The procedure leads to the self-consistent determination of the charge distribution in the nanostructure and the single-electron energy eigenstates of the Hamiltonian (1).

The phase diagrams discussed in our work concern primarily the total spin of the nanostructure, which is a sum of spin densities at individual sites, $S=\sum_{i=1}^{N}\langle s_{i}^{z}\rangle =\left(N_{↑}-N_{↓}\right)/2$. However, in order to search for the AFM phases with S=0, sublattice resolved magnetizations can also be discussed, being $S_{A,B}=\sum_{i=1}^{N_{A,B}}\langle s_{i}^{z}\rangle$, with $S=S_{A}+S_{B}$. The AFM phase would be then characterized by $S_{A}=-S_{B}$. To supplement the picture of magnetization distribution, the spins of triangles 1 and 2 (encircled with dashed lines in Figure 1), $S_{1,2}=\sum_{i\;\in \;\mathrm{triangle}\;1,2}^{}\langle s_{i}^{z}\rangle$, can also be discussed (note that the central dimer of carbon atoms lying on the symmetry axis of the structure is excluded from both triangles). The NM phase has vanishing spin density at all the sites forming the structure.

The next part of the paper presents the results obtained with the help of the above-described formalism. They concern primarily the ground-state phase diagrams of the heptauthrene nanostructure, both based on the total spin and including the AFM orderings. Moreover, spin distribution as well as the field dependence of the magnetizations and the single-electron energy states are discussed to accompany the investigation of the phases.

## 3. Results

The present section reports the results of our calculations, performed according to the methodology described in Section 2.

The general aim of the study is to examine the external electric field-induced transition between the singlet and the triplet spin state of the nanoflake. Therefore, the phase diagrams showing the spin state as a function of the external fields are of primary interest.

### 3.1. Magnetic Phase Diagrams

**Figure 2:**
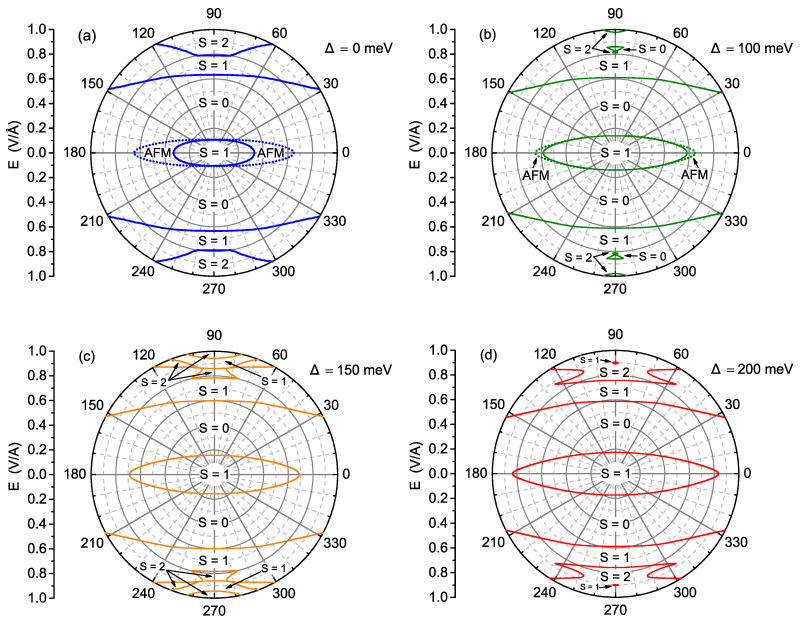
The ground-state magnetic phase diagram for the electric fields of magnitude not exceeding 1 V/Å and arbitrary in-plane orientation. Four values of exchange energy are selected: 0 meV (**a**), 100 meV (**b**), 150 meV (**c**), and 200 meV (**d**). The borders of phases with various values of total spin *S* are marked with solid lines. The dashed lines delimit the antiferromagnetic (AFM) and non-magnetic (NM) phase for S = 0.

In principle, the field components along the *x*- and *y*-axis can be considered as separate control variables, as the nanographene would be gated using two orthogonal pairs of gates. Figure 2 shows the phase diagrams in polar coordinates as a function of the strength and orientation of the electric field, for various values of possible exchange splitting energy in panels Figure 2a–d. The electric field magnitudes up to 1 V/Å are covered and the field parallel to the *x*-axis (see orientation in Figure 1b) corresponds to the polar angle equal to 0. The discrete rotational symmetry is clearly reflected in the phase diagrams. The solid lines delimit the phases with various values of the total spin of the nanoflake. In addition, within the phase with vanishing total spin, an antiferromagnetic orientation of spins is possible, and the boundary of this phase is marked with the dotted lines. In Figure 2a, the case of absent exchange splitting is shown. For a weak electric field, the ground state of triplet character, with S=1, is the stable state. If the field takes the *x* orientation, such state is most robust to the field increase; moreover, it first changes to the AFM state with S=0 and then, for a significantly stronger electric field, to the totally non-magnetic state. On the contrary, the *y* orientation of the electric field causes switching to the non-magnetic state for a much weaker field and severely limits the AFM ordering stability range. Further increase of the electric field with such orientation can also switch the state of the system back to S=1 and then to S=2, and this sort of behaviour is not seen for fields oriented along the *x*-axis in the studied range. In general, the stability range of the low-field state with S=1 has an elliptic-like shape, and the ellipse is strongly elongated along the *x*-direction. The presence of exchange splitting of Δ= 100 meV, shown in Figure 2b, significantly improves the stability of the triplet state when the field is oriented along the *x*-axis, at the cost of reducing the AFM stability range. When the field is acting in the *y*-direction, the AFM state is completely absent and the cross-over takes place directly to the totally non-magnetic state (also for a slightly higher field than for the case shown in Figure 2a). In contrast to the behaviour of the low-field S=1 state stability range, the boundary between the S=0 and high-field S=1 state is only weakly influenced (shifted to lower field). The state with S=1 present for stronger *E* fields becomes more stable at the cost of the state with S=2. Further increase in exchange splitting Δ (shown in Figure 2c,d) causes the stability range of S=1 low-field state to expand anisotropically, mainly along the $E_{x}$ direction, whereas the boundary between S=0 and S=1 state at higher $E_{y}$ fields is almost unchanged. The state with higher spin, S=2, tends to expand its stability range for stronger $E_{y}$ fields. All the described features are easily visible in Figure 2c for Δ= 150 meV and in Figure 2d for Δ= 200 meV. The phase diagrams shown in Figure 2 clearly indicate the role of the electric field orientation in the switching between the S=1 and S=0 state.

**Figure 3:**
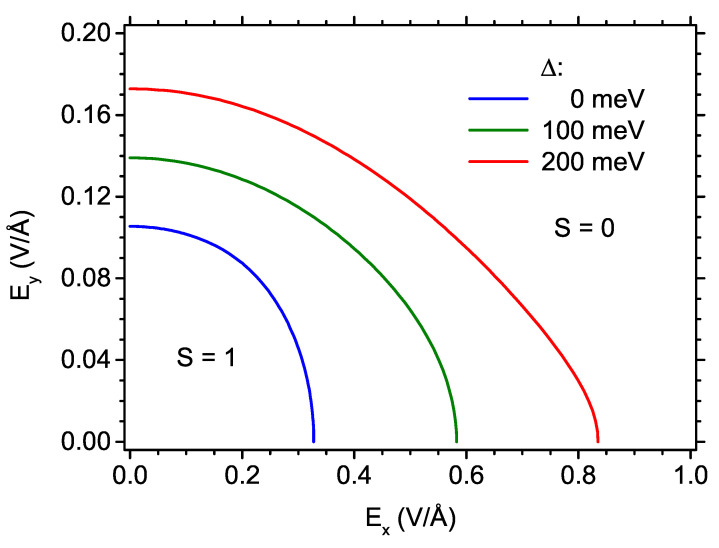
The phase diagram showing the borders between the state with S = 1 and the state with S = 0 as a function of the electric field along the *x*-direction and along the *y*-direction, for three values of exchange energy.

In order to investigate more precisely the ground-state phase diagram of heptauthrene nanographene, we plot the boundary between the low-field state with S=1 and the state with S=0 as a function of $E_{x}$ and $E_{y}$ component of the electric field in Figure 3. Three values of exchange splitting energy (Δ= 0, 100 and 200 meV) are compared. It is clearly visible that application of the electric field along the longest edge of the nanostructure (*y*-axis) permits the switching to the S=0 state using the field of approximately 0.1 V/Å, whereas the field applied only along the symmetry axis of the nanostructure has to be approximately three times stronger to produce the same effect when no exchange splitting is present. It is interesting that, applying some field in the *y*-direction, the critical value of $E_{x}$ necessary to switch the state can be tuned down to the desired value, so that both field components can lie approximately in the range of 0.1 V/Å. If Δ energy is increased, the critical field for switching increases much slower for the case of $E_{y}$ than in the case of $E_{x}$. However, even in the presence of large exchange splitting, the critical field $E_{x}$ can be reduced by application of $E_{y}$ only moderately higher than for the case of Δ= 0.

**Figure 4:**
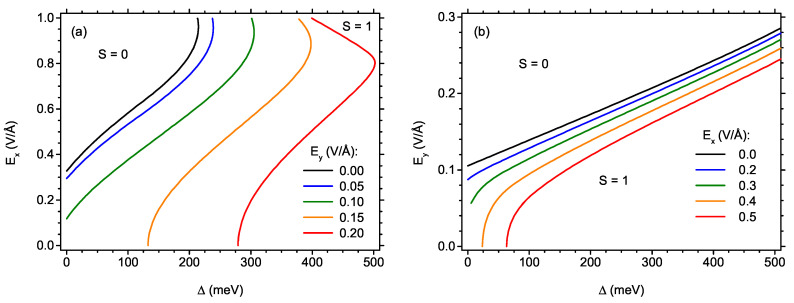
The phase diagram showing the borders between the state with S = 1 and the state with S = 0 as a function of the exchange energy and electric field along the *x*-direction (**a**) and along the *y*-direction (**b**), for various values of electric field along the other direction.

The influence of exchange splitting energy on the critical field for cross-over between the low-field S=1 state and S=0 state can be followed in details in Figure 4, where the critical field (either $E_{x}$ for Figure 4a or $E_{y}$ for Figure 4b) is plotted as a function of Δ. In Figure 4a, a few constant values of $E_{y}$ are selected, and it is visible how the increasing exchange splitting Δ very effectively stabilizes the S=1 state, whereas applying the *y*-axis-oriented electric field acts in the opposite direction, extending significantly the stability range of S=0 state. The behaviour of the critical field $E_{y}$ as a function of Δ can be tracked in Figure 4b for several values of $E_{x}$. Here, the exchange splitting also expands the range of S=1 state, but this time the critical field $E_{y}$ is much less sensitive to Δ than $E_{x}$ was in Figure 4a. For low $E_{x}$, the dependence of $E_{y}$ on exchange splitting is linear, while for stronger $E_{x}$ it linearises for larger values of Δ where the presence of the state with spin S=1 is enforced at low $E_{y}$. Figure 4 convinces readers that the critical field $E_{y}$ is much less sensitive to the presence of the exchange splitting than $E_{x}$.

**Figure 5:**
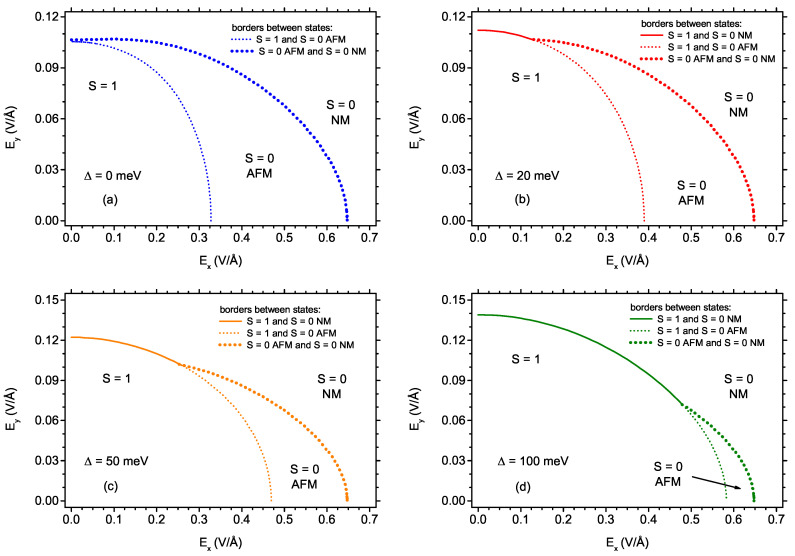
The phase diagram showing the borders between the state with S = 1 and the AFM and NM states with S = 0 as a function of the electric field along the *x*-direction and along the *y*-direction, for four values of exchange energy: 0 meV (**a**); 20 meV (**b**); 50 meV (**c**); and 100 meV (**d**). The solid lines delimit S = 1 and S = 0 NM state; the dashed lines delimit S = 1 and S = 0 AFM state; the dotted lines delimit S = 0 AFM and NM state.

As it has been already mentioned, the state with S=0 can be either of AFM nature, with non-zero spin densities of opposite signs at both carbon sublattices, or of a totally NM nature. The detailed stability range of the AFM state is plotted in Figure 5, where the stability ranges of S=1, S=0 AFM state and S=0 NM state are marked. The solid lines depict the border directly between the S=1 and S=0 NM state, while the dashed lines mark the transition between S=1 and S=0 AFM and the dotted lines denote the border between S=0 AFM and NM state. The panels Figure 5a–d are prepared for the increasing values of the exchange splitting, ranging from 0 (Figure 5a), through 20 meV (Figure 5b), 50 meV Figure 5c and up to 100 meV (Figure 5d). It can be noticed that the border between S=0 AFM and NM state is insensitive to the presence of exchange splitting and only the stability range of S=1 phase expands when Δ increases, thus replacing the S=0 AFM phase at low fields $E_{x}$. For Δ values exceeding 100 meV, the AFM phase is completely displaced by S=1 phase. In the absence of the exchange splitting, the stability range of AFM phase is as wide as for the S=1 phase when $E_{y}=0$ and the electric field is applied along the *x*-direction. If the electric field is applied along the *y*-axis, the stability range of AFM phase is marginal and the borders of S=1, S=0 AFM, and S=0 NM states almost touch at $E_{x}=0$. If some exchange splitting is switched on, a sort of triple point emerges in the phase diagram. Namely, at low $E_{x}$, increasing the field $E_{y}$ causes switching directly from S=1 to S=0 NM, while for stronger $E_{x}$ the S=0 AFM state emerges in between the previously mentioned states. The position of the triple point, in which S=1, S=0 AFM, and S=0 NM states are stable, slides along the AFM-NM border, as it can be followed in the further panels of Figure 5c,d.

**Figure 6:**
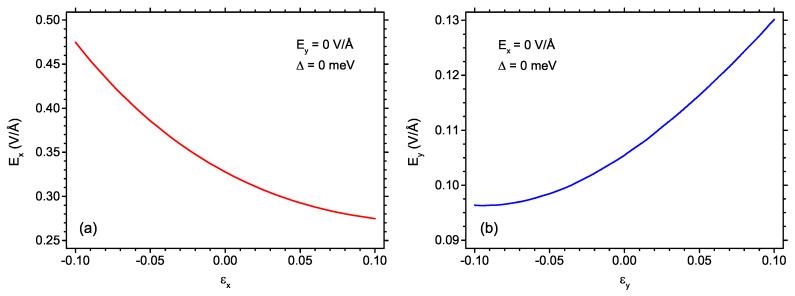
The dependence of the critical electric field along the given direction on the strain along the same direction: (**a**) *x* direction and (**b**) *y* direction, in the absence of other fields. The critical field corresponds to switching between S = 1 and S = 0 state.

The results presented above assume lack of strain in the studied structure. However, the deformation of heptauthrene nanoflake with respect to the ideal geometry can arise, either due to the interaction with the substrate or as a consequence of the presence of an electric field. In order to assess the strain effect on the critical fields necessary for switching between the S=1 and S=0 state, we have simulated the effect of uniaxial strain in the structure either along the *x*- or along the *y*-direction. The corresponding results are shown in Figure 6. For the plots, the absence of exchange field is assumed, and the electric field is applied only along one direction: *x*-direction for Figure 6a and *y*-direction for Figure 6b. The negative sign of strain corresponds to compressive strain, whereas the positive sign introduces tensile strain; the considered magnitude does not exceed 0.1. For both studied field and strain directions, the dependence of the critical field on strain follows a non-linear convex function. The compressive strain along *x*-directions tends to increase the critical field $E_{x}$ in quite a visible manner, whereas the effect of the tensile strain, consisting in reduction of the critical field, is much less pronounced. The situation for electric field and strain along the *y*-direction is quite the opposite. The tensile strain $\epsilon_{y}$ causes the field $E_{y}$ to increase, whereas the effect of the compressive strain is to limit the critical field (the latter effect is less pronounced than the former one). The overall effect of the strain on the critical field along the *y*-direction is much weaker than for the case of the *x*-direction. Let us emphasize that the range of strains considered in the calculations reported in Figure 6 is quite large. The issue of electrostrictive deformation of nanographenes has been subject to a Density Functional Theory-based study [82] (including a benzene ring and phenanthrene molecule). It has been predicted that the C-C bond deformation in hydrocarbon structures is rather limited (below 0.2%) even in the field of 1 V/Å. Therefore, the magnetic phase diagram predicted on the basis of the model not assuming the presence of the strain seems to provide a reliable physical picture.

### 3.2. Spin Distribution and Energy States

**Figure 7:**
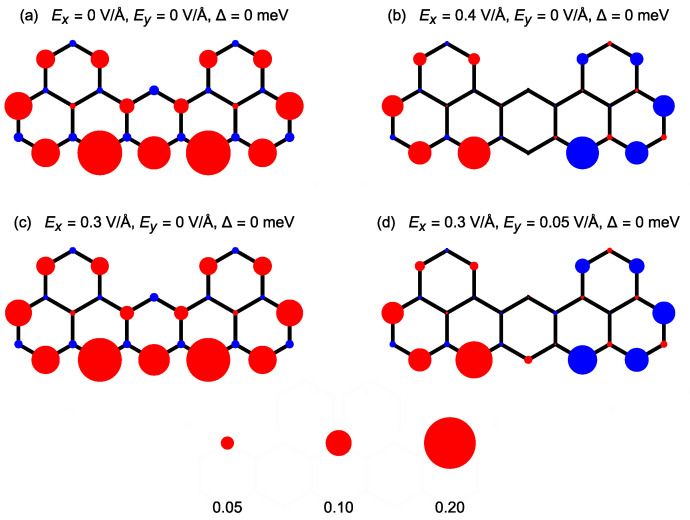
Spin density distribution across the nanostructure for various values of the electric field, in the absence of the exchange energy: $E_{x}\;=\;0$, $E_{y}\;=\;0$ (**a**); $E_{x}\;=\;0.4$ V/Å, $E_{y}\;=\;0$ (**b**); $E_{x}\;=\;0.3$ V/Å, $E_{y}\;=\;0$ (**c**); $E_{x}\;=\;0.3$ V/Å, $E_{y}\;=\;0.05$ V/Å (**d**). Red and blue colours mark two antiparallel spin orientations. The radius of the circle is proportional to the spin density (the scale is shown at the bottom of the plot).

**Figure 8:**
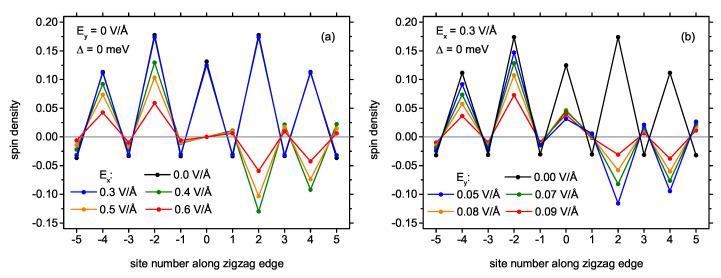
Spin distribution along the longest zigzag edge of the nanostructure in the absence of the exchange energy, for electric field $E_{y}\;=\;$ 0 and a few selected values of $E_{x}$ (**a**) and for electric field $E_{x}\;=\;$ 0.3 V/Å and a few selected values of $E_{y}$ (**b**). Lines are guides to eyes only.

**Figure 9:**
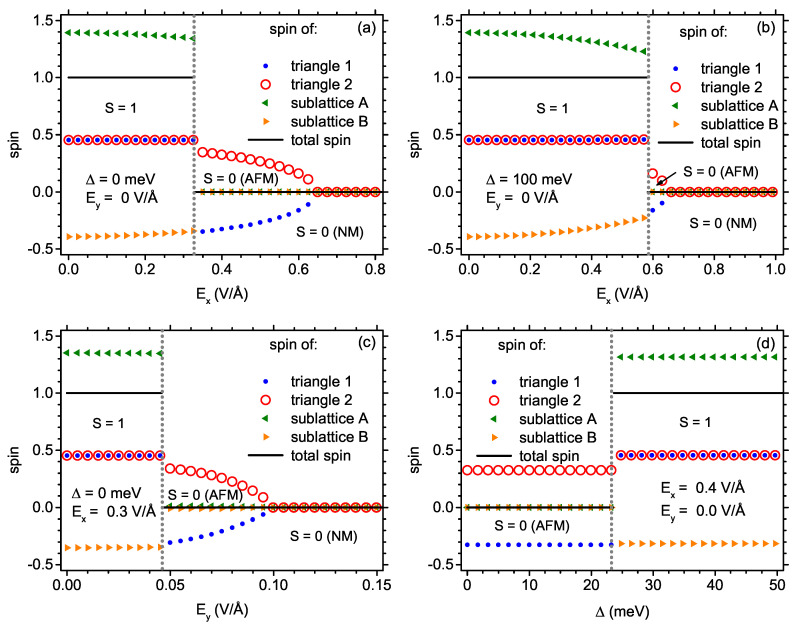
Total spin of the nanostructure and spins of sublattices and triangles as a function of: (**a**) electric field $E_{x}$ for $E_{y}\;=\;0$ and Δ = 0; (**b**) electric field $E_{x}$ for $E_{y}\;=\;0$ and Δ = 100 meV; (**c**) electric field $E_{y}$ for $E_{x}\;=\;0.3$ V/Å and Δ = 0; (**d**) exchange energy Δ for $E_{x}\;=\;0.4$ V/Å and $E_{y}\;=\;0$. Vertical dotted lines mark the points at which the state changes.

After analysis of the phase diagrams, it is interesting to investigate the spin density distribution across the nanostructure for the particular magnetic states (see also Figure 2c in Ref. [69]). As it follows from the above discussion, the state in the absence of the electric and magnetic field is a S=1 state. The spin distribution for this case is plotted in Figure 7a. It follows that spin density is concentrated at the outer atoms of the longest zigzag edge of the structure, in concert with a general expectation that this kind of edge favours the magnetic polarization [42]. Moreover, the state is actually of a ferrimagnetic nature, as sites belonging to both sublattices show the opposite sign of magnetic polarization (with very weak polarization of the minority sublattice). The spin distribution along the zigzag edge can be further traced in details in Figure 8 (where site number equal to 0 corresponds to the carbon atom belonging to the central dimer connecting two triangles—see the site labels in Figure 1b). The case of Δ = 0 and $E_{y}$ = 0 is shown in Figure 8a. When $E_{x}$ field is applied (like $E_{x}=$ 0.4 V/Å in Figure 7b), the nanoflake switches to the AF state with total spin equal to 0. In addition, in this case, the spin density is distributed dominantly along zig-zag edge, but this time it is much less uniform in magnitude, as it can be confirmed by Figure 8a. In particular, there is no noticeable spin density at the central carbon dimer connecting the two triangles. Therefore, spin density at both triangles is well separated. The application of the field along the *x*-direction does not spoil the symmetry of the spin density distribution with respect to the nanostructure symmetry axis. The magnitudes of spin density at individual sites are reduced with respect to the triplet case. Of course, further increase in $E_{x}$ leads to continuous reduction of the spin densities and, then, to the totally non-magnetic state with no local spin polarization (as illustrated in Figure 9a). Let us stress that the local spin distributions for the state with S=1 are insensitive to the electric field up to the critical field; on the contrary, the spin densities for AF state change in a continuous manner with the field. The effect of switching the spin state from S=1 to S=0 under the influence of the electric field applied along the *y*-direction is shown in Figure 7c,d and also in Figure 8b, at the fixed electric field of $E_{x}=$ 0.3 V/Å and Δ = 0. The whole process progresses in analogous manner as in the case of switching with the field oriented along the *x*-direction.

The behaviour of the individual components of the total magnetization also deserves separate interest. First, the total spin can be considered as a sum of spin densities at carbon sites belonging to two sublattices: the sublattice A with 15 atoms and sublattice B with 13 atoms. On the other hand, the total magnetization is a sum of the magnetizations of both triangles forming the nanostructure and the magnetization of the central dimer interconnecting the triangles (where, for practical analysis, the dimer magnetization could be omitted). Let us remind readers that the schematic view of the nanostructure is shown in Figure 1. The dependence of the total magnetization as well as its mentioned components on the electric field $E_{x}$ in the absence of exchange splitting and electric field $E_{y}$ can be followed in Figure 9a. Below the critical field $E_{x}$, the total magnetization is equal to 1 (as required by S=1 state) with two triangle magnetizations parallel and slightly lower than 1/2 (equal to 0.453), as the central dimer spin is not included. The majority sublattice takes the magnetization of 1.393, whereas the minority one has the total magnetization of −0.393. The values give flavour of the ferrimagnetic character of S=1 state. If the field $E_{x}$ is increased below the critical field, the triangle magnetizations remain almost constant, whereas the sublattice components decrease slightly in their magnitudes, keeping the total magnetization equal to 1. At the critical field, the total magnetization value and the component values change discontinuously. For $E_{x}$ exceeding the critical field, both sublattices have magnetizations equal to zero, whereas the magnetizations of triangles become opposite in orientation and keep non-zero magnitude of 0.354 (which is slightly reduced with respect to the value taken at S=1 state). This magnitude is then continuously reduced when $E_{x}$ increases and, finally, it vanishes when the system switches to the S=0 NM state. The analogous dependence of the magnetizations on $E_{x}$ is shown in Figure 9b in the presence of the exchange splitting equal to 100 meV and in the absence of the $E_{y}$ electric field. The whole physical picture is essentially similar to the case shown in Figure 9a and only the critical field for switching between S=1 and S=0 AFM state is increased by the presence of Δ. The critical field for transition between AFM and NM state is unchanged. The effect of increasing electric field $E_{y}$ on the magnetizations in the presence of $E_{x}=$ 0.3 V/Å and in the absence of exchange field is shown in Figure 9c. In the low-field state S=1, the sublattices take the magnetizations 1.351 and −0.351, respectively, while both triangles indicate the magnetization of 0.454. Both sublattice and triangle magnetizations remain fairly constant when the electric field $E_{y}$ is varied. For $E_{y}$ exceeding the critical value, the switching to S=0 AFM state occurs. Again, the total sublattice magnetizations vanish and the triangle magnetizations continuously decrease to reach zero at the transition from AFM to NM state. The process of switching from S=0 AFM to S=1 state by increasing the exchange splitting Δ in the presence of the electric field $E_{x}=$ 0.4 V/Å can be tracked in Figure 9d. It is visible that all the discussed magnetizations remain constant when the exchange splitting is varied within the stability range of a given state. For AFM state, the total sublattice magnetizations vanish and both triangles show the opposite sign of magnetizations with the magnitude of 0.326, therefore significantly reduced with respect to the value of 1/2. For S=1 state above the critical exchange splitting energy, the majority sublattice takes the magnetization of 1.316 and the minority one of −0.316, adding up to S=1. Both triangles exhibit parallel magnetizations slightly lower than 1/2 (equal to 0.455).

**Figure 10:**
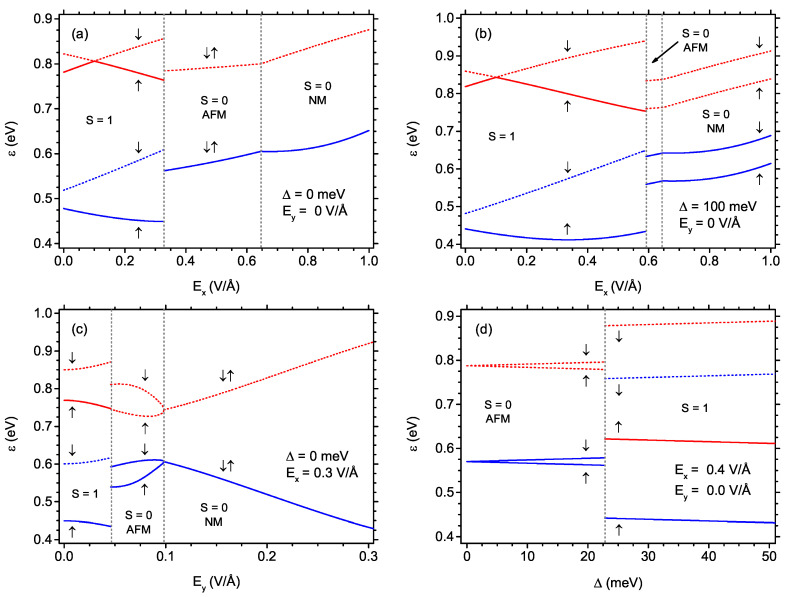
Single-electron eigenenergies as a function of: (**a**) electric field $E_{x}$ for $E_{y}\;=\;0$ and Δ = 0; (**b**) electric field $E_{x}$ for $E_{y}\;=\;0$ and Δ = 100 meV; (**c**) electric field $E_{y}$ for $E_{x}\;=\;0.3$ V/Å and Δ = 0; (**d**) exchange energy Δ for $E_{x}\;=\;0.4$ V/Å and $E_{y}\;=\;0$. Vertical dotted lines mark the points at which the state changes. The solid lines denote the states occupied by electron, while the dashed lines mark empty states. The arrows indicate the spin direction of the state. The blue color marks the lower energy and the red colour the higher energy state for the given spin orientation. Only two occupied states highest in energy and two empty states lowest in energy are shown.

The magnetic properties of the nanostructure in question can be related to the behaviour of the individual, single-electron energy states under the influence of the external field. Figure 10 presents the dependence of the energies of the highest occupied and lowest unoccupied energy states on the external electric field or exchange energy. In all the panels, solid lines mark the states occupied by electrons, whereas dashed lines refer to the unoccupied states. Moreover, the blue color is for lower energy for given spin orientation, while the red one denotes the higher energy for given spin orientation. The arrows mark the spin orientation assigned to the given energy state (two arrows pointing in opposite directions correspond to spin-degenerate state). The influence of the electric field $E_{x}$ in the absence of $E_{y}$ and Δ can be followed in Figure 10a. In the S=1 state at low electric field $E_{x}$, two states with spin up are occupied and two states with spin down are empty. Please note that all the states with energy lower than plotted in Figure 10a are occupied by the electrons (with equal number of non-degenerate states with spin up and spin down). At the critical electric field $E_{x}$, the energies change in discontinuous manner and the states become spin-degenerate (even though the state is AFM, not NM). At the point of further transition from S=0 AFM to NM state, the energies are continuous (with just a jump in the derivative). It must be noticed that all the plotted energies originate from the self-consistent diagonalisation of the mean-field Hubbard Hamiltonian (1) at the half-filling condition. Even though they represent single-particle energy states, the eigenvalues would change after adding or subtracting the electrons from the system (i.e., charge doping). Moreover, the discontinuous behaviour of the Hamiltonian eigenvalues does not imply the discontinuity of the total energy (being the sum of the energies of the occupied states); the total energy is continuous at the transition from S=1 to S=0 state—see the similar discussion in our work Ref. [53]. The panel Figure 10a can be correlated with the plot in Figure 9a showing the behaviour of the magnetizations in the same conditions. The analogous sequence of transitions as in Figure 10a can be tracked in Figure 10b in the presence of finite exchange energy amounting to 100 meV. Below the critical field $E_{x}$ in the S=1 state, the situation is similar to the case of Δ= 0. At the critical field when the state switches from S=1 to S=0, the energies vary discontinuously, but, in the S=0 AFM state, they remain spin-splitted (what results from Δ>0). This splitting is also visible for the S=0 NM state. Two states of lower energy (spin-up and spin-down) are occupied, while two states of higher energy become empty. The plot Figure 10b reflects the conditions for which we prepared Figure 9b.

The effect of increasing electric field $E_{y}$ in the absence of exchange energy Δ and for finite field $E_{x}=$ 0.3 V/Å can be followed in Figure 10c. The low-field state is S=1, with significant spin-splitting of the energy states (with both occupied states having up orientation of spin and two empty spin-down states). When the state switches to S=0 AFM, the energy eigenvalues exhibit discontinuous change; two states with opposite spin and lower in energy are occupied, two analogous states with higher energies are empty. The spin-splitting of the states continuously decreases when $E_{y}$ is increased and the states become spin-degenerate exactly at the field when the system shows cross-over to the S=0 NM state. In the NM state stability range, the states remain spin-degenerate. The described behaviour can be confronted with the behaviour of magnetizations in Figure 9c. The influence of the increasing exchange energy Δ is illustrated in Figure 10d for the finite field $E_{x}=$ 0.4 V/Å. The state at Δ=0 is S=0 AFM state and the energy states are spin-degenerate at that point. Increasing the exchange energy causes the linear increase in energy difference between spin-up and spin-down states (with one pair of states occupied and one pair empty). At the critical value of Δ, where S=1 state emerges, the energies jump discontinuously and the spin-splitting (the energy difference between the spin-up and spin-down state) increases significantly. The behaviour of magnetizations for analogous conditions is shown in Figure 9d. It can be observed that, at S=0, the AFM state, the energy states can be either spin-degenerate or spin-splitted, depending on the interplay of Δ and both components of the electric field. In S=0 NM state, the energy eigenstates are spin-splitted only if Δ>0 and, for S=1, the spin-splitting is always present.

## 4. Discussion

After presentation of our computational predictions of the magnetism in the heptauthrene molecule and its sensitivity to the external electric field, some related issues might be mentioned below and some future research directions can be drawn.

The first issue would be related to the reliability of the model used for computations of the magnetic phase diagram. In this context, it might be mentioned first that the Hubbard model in mean-field approximation has been used in Ref. [69] to simulate the density of states distribution across the heptauthrene nanostructure, which has been successfully compared with the results of scanning tunneling microscopy experiment. In addition, an identical model formalism has been utilized with analogous aim in other studies related to molecular graphene nanostructures with zigzag edges and the consistency between its outcome and the scanning tunneling microscopy results has been found [22,23,37,68]. In particular, the consistency of exchange interaction energies between the Hubbard model-based calculations and the scanning tunneling spectroscopy experiment was found [37]. Therefore, the mean-field Hubbard model which we use in our study is a reliable tool in characterization and prediction of magnetism in graphene nanostructures.

It might be mentioned that the presence of the in-plane electric field might cause some deformation of the nanostructure, constituting the manifestation of the electrostriction. Such effect has been studied theoretically [82] and only a very limited influence of the fields up to 1 V/Å has been found for the hydrocarbon molecules. This fact had been attributed to the absence of dangling bonds (as the effect in identical structures but without hydrogen atoms saturating the dangling bonds was significantly enhanced). Much larger electrostrictive deformation has been found in carbon nanotube structures [82,83], reaching the value of about 10% for the fields of the order of 0.4 V/Å [83]. However, carbon nanotubes constitute very different systems than planar nanographenes. As a consequence, we do not expect the fundamental influence of the electrostrictive deformation of the graphene nanoflake on the calculated magnetic phase diagram. It can be also noted that the detailed deformation of the nanoflake might also be influenced by its interaction with the substrate.

In the context of application of the electric field to the studied structure, it should be recalled that characterization of nanographenes with scanning tunneling microscopy techniques involves the interaction with the electric field in the vicinity of the tip [84]. The tip-related electric field may reach high values (of the order of V/Å) and extends in a significant range around the tip [84]. Therefore, it also can, in principle, deform the studied nanostructure.

Let us comment that the parameter Δ which we introduce in our theoretical model to account for the spin-dependent energy splitting can originate not from the external magnetic field itself but primarily from the exchange field coming from the ferromagnetic substrate. We mention here that the splitting of S=1 energy state slightly less that 2 meV was measured in heptauthrene at the external magnetic field of 3 T [69]. Taking into account this result, the exchange splitting having the significant impact on the phase diagram might be expected rather as a consequence of interaction with the magnetic substrate [85]. Let us note that the values of exchange parameter in the Hamiltonian of graphene on magnetic insulators are predicted to reach even hundreds of meV [78]. It needs to be emphasized that the value of Δ parameter in Hamiltonian (1) should not be regarded as equivalent directly to the value of spin-splitting of the individual energy state for S=1 (as visible in Figure 10), as the energy levels come from the diagonalization of the full Hamiltonian including all the contributions.

An important issue from the point of view of experimental validation of the predictions and of the possible applications is the stability of the predicted spin polarization (focused mainly at the edge of the nanostructure). The stability might be discussed both in the context of the thermal excitations at finite temperature [86] and the possible influence of the electrodes providing the electric field on the magnetic edge ordering [87]. Nevertheless, the experimental results of Ref. [74] can be recalled here, as the room-temperature measurements of the energy gap in nanoribbons revealed the splitting attributed to the presence of the magnetic ordering. Moreover, in heptauthrene nanostructure investigated in our paper, the Kondo resonance has been identified by means of the scanning tunneling spectroscopy [69], which provided sound confirmation of the presence of the spin triplet state. Furthermore, the possibility of influencing the magnetic state by addition of a hydrogen atom found in Ref. [69] also proved the existence of magnetic orderings in the studied nanosystem. In addition, the issues with potential bistability of edge magnetization in graphene nanostructures in the presence of the electric field can be mentioned [88]. All these factors would inspire some further investigations.

The application of the in-plane electric field to the discussed nanostructure would require gating of the structure. The small in-plane extension of the nanostructure should limit the screening of the in-plane electric field (the factor not included in the present study), thus facilitating the switching process. On the other hand, our study was performed for the assumption of charge neutrality, meaning the half-filling of the energy levels of the nanostructure with a fixed number of electrons (equal to the number of carbon atoms). Coupling to the gates would cause electron tunneling process and vary the electron number in the structure, resulting in charge doping. Charge doping would also result from the interaction with the substrate. This factor would also shape the magnetic phase diagram and would inspire separate study aimed at evaluating its importance.

The geometry of the graphene nanosystem placed between the gates would also turn the attention to the transport properties and the possibility of tuning them by altering the spin state. The structure of heptautherene would remind readers of a sort of double quantum dot with switchable spin states of both triangles. The transport properties of various graphene-based quantum dots and analogous systems attracted some theoretical attention so far [89,90,91,92,93,94] and certainly this direction of research would also include the heptauthrene nanosystem.

Finally, the possible extensions of our theoretical model for the studied molecule might include study of the influence of proximity-induced spin-orbit coupling [95] on the phase diagram of the system, permitting the presence of non-collinear magnetic phases (see, for example, Refs. [96,97,98]). This factor would possibly enrich the magnetic phase diagram and provide an additional route to manipulation of magnetism with the electric field.

## 5. Conclusions

In the paper, we have investigated the influence of the external electric field and magnetic exchange field on the magnetic phase diagram of heptauthrene nanostructure. The study was inspired by successful synthesis of the atomically precise nanostructure [69] and scanning tunneling microscopy characterization of its electronic states, consistent with the Hubbard model-based calculations. The study Ref. [69] confirmed the presence of the triplet ground state with S=1 in the absence of the external fields. The above-mentioned facts serve as motivation for characterization of the magnetic phase diagram of heptauthrene using a mean-field based Hubbard model and for studying the possibility of exploiting the magnetoelectric phenomena to control the magnetic state with the in-plane electric field. Such possibility is of importance for the spintronic applications.

The possibility of switching the nanostructure to the state with S=0 by applying the electric field even of the order of 0.1 V/Å has been predicted. The pronounced directional anisotropy of the critical electric field has been found (with the lowest field needed to change the state when applied along the longest edge of the structure). Moreover, the possibility of lowering the critical field in one direction when applying a constant field in the perpendicular direction has been postulated. The presence of AFM state (with the spins of two triangles pointing in the opposite directions) was predicted within the stability range of the S=0 phase. The exchange field (in-plane magnetic field) was found to stabilize the S=1 state at the cost of the S=0 state. Whilst the spin densities within S=1 state are predicted to be insensitive to the electric fields, they decrease continuously if the field is increased in the stability range of the S=0 AFM state. The effect of the uniaxial strain on the critical electric fields has been sketched, with more pronounced importance of strain along the *x*-direction.

The calculations are based on the mean-field Hubbard model, capturing the essence of electronic correlations responsible for the magnetism in graphene nanostructures and used to interpret the experimental results for nanographenes [22,23,37,68] and other systems [74]. Therefore, it can be regarded as a useful and reliable tool to predict the nature of the magnetic behaviour of the graphene nanostructures [99], which can be effectively characterized experimentally using scanning tunneling microscopy techniques. Moreover, the paper would motivate further studies, for example employing density functional theory methods, focused on the heptauthrene nanostructure deposited on particular substrates and the detailed behaviour of such system in the external field. 

## Data Availability

All the obtained results of computations were presented in plots contained in the paper.

## Abbreviations

The following abbreviations are used in this manuscript:

AFM	antiferromagnetic
NM	non-magnetic
